# Long non-coding RNAs and their potential function in response to postharvest senescence of *Sparassis latifolia* during cold storage

**DOI:** 10.1038/s41598-023-46744-2

**Published:** 2024-01-07

**Authors:** Mengting Weng, Di Zhang, Hongyu Wang, Chi Yang, Hongyi Lin, Yanfang Pan, Yanquan Lin

**Affiliations:** 1 Institute of Edible Mushroom, Fujian Academy of Agricultural Sciences, Fuzhou, 350014 China; 2 National and Local Joint Engineering Research Center for Breeding & Cultivation of Featured Edible Mushroom, Fujian Academy of Agricultural Sciences, Fuzhou, 350014 China; 3 Institute of Food Science and Technology, Chinese Academy of Agricultural Sciences, Beijing, 100193 China

**Keywords:** Biochemistry, RNA

## Abstract

Long non-coding RNAs (lncRNAs) have been shown to play crucial roles in response to aging processes. However, how lncRNAs regulate postharvest senescence of *Sparassis latifolia* (*S. latifolia*) with oriented polypropylene (OPP) film packing during cold storage remains unclear. In this study, we performed RNA-seq using the fruiting bodies of *S. latifolia* stored at 4 ℃ for 0, 8, 16 and 24 days after harvest, and profiled the lncRNA and mRNA transcriptome, respectively. In total, 1003 putative lncRNAs were identified, and there were 495, 483 and 162 differentially expressed (DE) lncRNAs, and 3680, 3941 and 1870 differentially expressed mRNAs after 8, 16 and 24 days of storage, respectively, compared to 0 day of storage. Target genes of differentially expressed lncRNAs were found to significantly associate with carbon and energy metabolism, response to abiotic stimulus, amino acid biosynthesis and metabolism, and protein synthesis and transcription. In addition, DE-lncRNA-mRNA co-expression networks in response to aging stress were also constructed. Taken together, these results confirm the regulatory role of lncRNAs in postharvest senescence of *S. latifolia* and will facilitate for improving preservation method.

## Introduction

*Sparassis latifolia* (*S. latifolia*) Y.C. Dai et Z. Wang (Sparassidaceae, Polyporales, Agaricomycetes)^1^, also called cauliflower mushroom, exhibits therapeutic value mainly because of the high β-glucan content^2,3^. To 2021, the total fresh fruit production of *S. latifolia* has exceeded 25 tons/d in Chinese factories^4,5^. Fujian province is one of the main areas producing *S. latifolia*^5^. Fresh fruiting bodies of *S. latifolia* are sold all over the country under low temperature with OPP film packaging in a large part. Nevertheless, fruiting bodies will age even deteriorate with the prolonging of postharvest storage and transportation. To achieve the longer time storage and longer distance transportation, and maximum commercial value of *S. latifolia*, its necessary to understand internal mechanism of postharvest changes.

Postharvest senescence of mushroom is a complex process accompanying with water loss, texture softening, nutrition loss, browning, flavor changes, and microbial attack, which seriously reduces its commercial worth^6–8^. In terms of studies on the postharvest changes of mushrooms, a few technologies have been applied to explore the related pathways, proteins and genes, including genome and transcriptome sequencing, proteomic and metabolomic analysis^9,10^. Postharvest age-related signaling pathways of *Volvariella volvacea* mainly including protein synthesis and transcription, fatty acid biosynthesis and metabolism, amino acid biosynthesis and metabolism and energy metabolism have been found using proteomics study^11^. Using transcriptome sequencing, the genes such as cell wall-related enzymes involved in the loss of quality of *Lentinula edodes* postharvest fruiting bodies were predicted^9^. With the rapid development of RNA-seq library construction methods and high-throughput sequencing technology, more and more studies have unveiled that not only mRNAs but also long non-coding RNAs are engaged in a wide range of biological processes, such as controlling flowering period^12^, influence pollen development^13^, governing cotton fiber development^14^, as well as responding to stress-induced aging^15^.

Long non-coding RNAs (lncRNAs) are a class of transcripts that have more than 200 base pair and poor proteins coding potential, which were produced by RNA polymerases (Pol I, II, III, IV, and V) in plants^16–18^. According to their location relative to protein-coding genes, lncRNAs can be classified into antisense, intronic, promoter, and intergenic lncRNAs^19^. LncRNAs participate in important age-related signaling pathways via regulation the expression of target genes in cis or trans modes^20^. A large number of differentially expressed lncRNAs were identified, which target genes were coding proteins involved in age-related signaling pathways, like enzymes associated with cell wall degradation, lipid peroxidation, and secondary metabolism^21^. Some DE-lncRNAs, such as lncRNAs MSTRG.31014.21 and MSTRG.31014.36, which are possibly involved in flag leaf senescence of rice could regulate the abscisic-acid biosynthetic gene BGIOSGA025169 (OsNCED4) and BGIOSGA016313 (NAC family)^22^. LncRNA AT5G01595 might regulate the expression of FER1during leaf senescence in *Arabidopsis*^15^. Although lncRNAs have crucial functions and effects on the course of aging, the functions of lncRNAs in aging process of *S. latifolia* are not yet well validated during postharvest storage.

We have previously studied the physicochemical indexes, including the weight loss, color difference, total sugar, total protein, total phenol, β-glucan and ergosterol content of *S. latifolia* stored at 4 ℃ packed with OPP films, and found that the eighth day was an important time point^23^. So, in this study, four time points, namely 0, 8, 16 and 24 days, were selected for subsequent transcript analysis. We tried to investigate the roles of postharvest senescence-responsive lncRNAs in *S. latifolia*. Therefore, lncRNAs in *S. latifolia* were systematically identified for the first time, and differentially expressed lncRNAs as well as their target differentially expressed mRNAs at different storage times were obtained. Then, the function of differentially expressed target mRNAs was predicted. We also constructed lncRNA-mRNA coexpression networks, through which may find lncRNAs with active roles in postharvest senescence of *S. latifolia*. Overall, this study may verify role of lncRNAs in postharvest senescence and provide clues to prolong the fresh-keeping period of *S. latifolia*.

## Results

### Identification and characterization of lncRNAs and mRNAs

In the present study, RNA-seq of 12 *S. latifolia* samples were performed and obtained 68,936,558–111,554,056 clean reads (9.59–16.61 GB clean bases) with a Q30 of 92.28–94.22%. There were 71.20–90.25% of the clean reads in each library mapped to the *S. latifolia* reference genome, of which the unique alignment rates ranged from 56.21 to 85.38%. The detailed information of RNA-seq was shown in Table 1.

**Table 1:** The detailed information of RNA sequencing.

Groups	sample	Raw reads	Clean reads	Clean bases	Error rate (%)	Q20 (%)	Q30 (%)	GC content (%)	Total mapped (%)	Uniquely mapped (%)
Group A (0 day)	S_latifolia0_1	81,673,678	80,341,990	10,981,199,997	0.0266	97.35	92.74	53.86	90.09	84.4
	S_latifolia0_2	76,240,266	75,006,300	10,346,303,204	0.0266	97.34	92.74	53.36	88.61	83.3
	S_latifolia0_3	71,432,626	70,972,686	9,886,480,429	0.0271	97.12	92.28	53.58	90.25	85.38
Group B (8 day)	S_latifolia8_1	110,148,518	111,554,056	16,606,619,798	0.0251	97.78	94.22	51.31	79.08	60.26
	S_latifolia8_2	92,131,416	92,630,010	13,775,812,603	0.0252	97.83	94.14	53.46	73.21	56.21
	S_latifolia8_3	110,305,723	106,147,706	15,594,698,808	0.0253	97.68	94.08	50.69	78.77	64.81
Group C (16 day)	S_latifolia16_1	119,577,055	110,177,462	15,867,147,223	0.0252	97.79	94.16	52.29	87.49	82.15
	S_latifolia16_2	98,227,591	92,713,360	13,497,287,889	0.0251	97.79	94.3	50.88	87.24	82.21
	S_latifolia16_3	78,099,419	72,966,626	10,546,011,508	0.0261	97.35	93.32	52.49	87.74	82.53
Group D (24 day)	S_latifolia24_1	79,223,008	78,099,136	10,762,177,065	0.0268	97.27	92.57	53.16	75.87	67.59
	S_latifolia24_2	74,215,258	73,443,512	10,171,642,562	0.027	97.16	92.35	53.19	84.95	71.71
	S_latifolia24_3	69,988,408	68,936,558	9,588,239,519	0.0269	97.2	92.43	53.17	71.2	57.68

**Figure 1:**
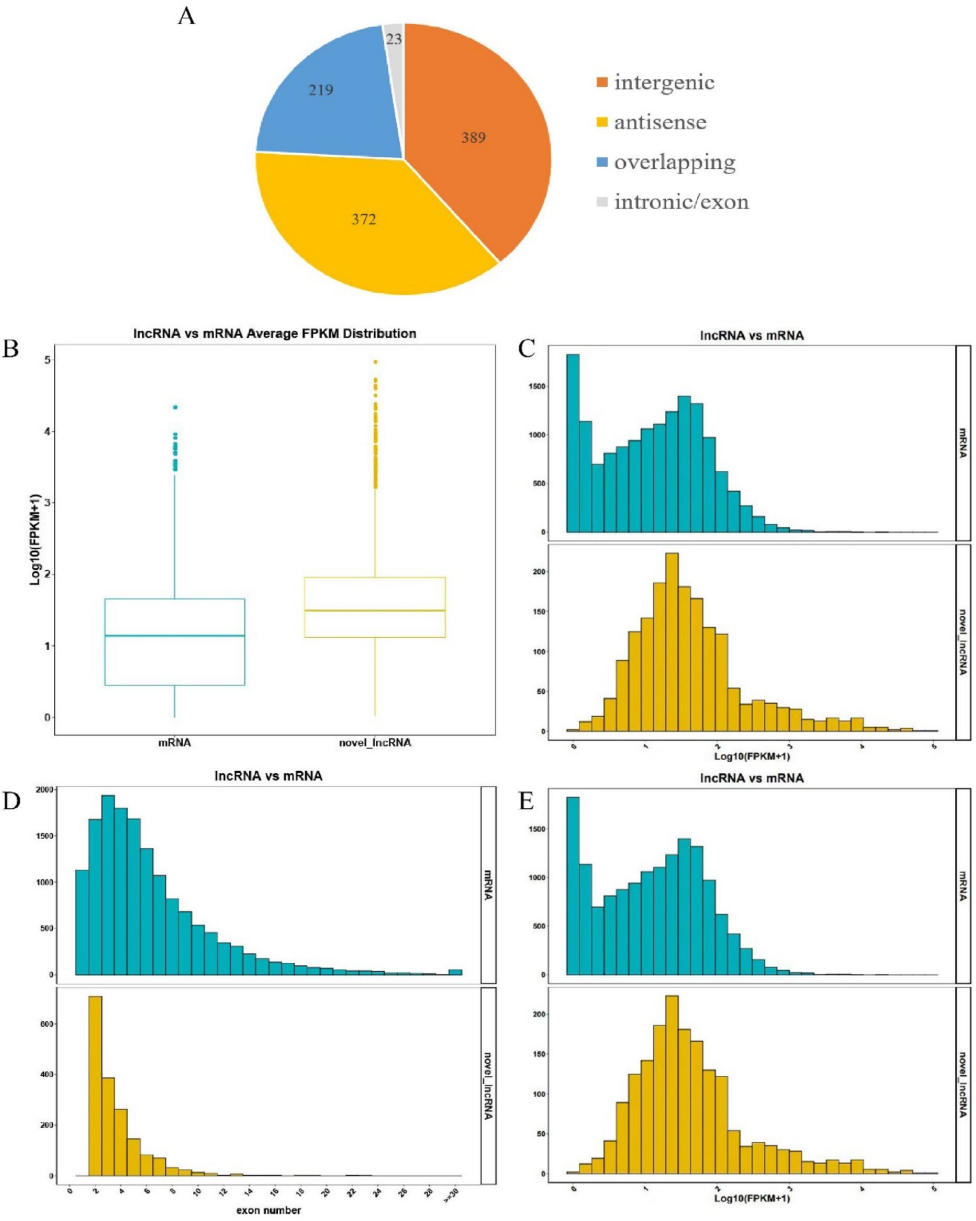
Classification and characterization of the identified lncRNAs. (**A**) Composition of multiple types of lncRNAs (**B**), (**C**) Expression of lncRNAs and mRNAs (**D**) Distribution diagram of exon (**E**) Distribution diagram of transcript length.

1003 lncRNAs were identified, which were all identified as novel lncRNAs because of lacking of lncRNA information for *S. latifolia*. In addition, we classified these lncRNAs into four classes: 389 intergenic lncRNAs (38.78%), 372 antisense lncRNAs (37.09%), 23 intronic/exon lncRNAs (2.29%), and 219 overlapping lncRNAs (21.83%) (Fig. 1A).

To unravel the correlation between lncRNAs and mRNAs involved in the senescence, we also identified 13,103 mRNAs in this study. The mRNAs identified in this study had more exons than lncRNAs. Most lncRNAs had fewer than 15 exons, but a part of mRNAs had more than thirty exons. In addition, most lncRNAs had shorter transcript length compared with mRNAs. However, the highly expressed lncRNAs were more than mRNAs (Fig. 1B–E).

### Differential expression analysis of lncRNAs and mRNAs

**Figure 2:**
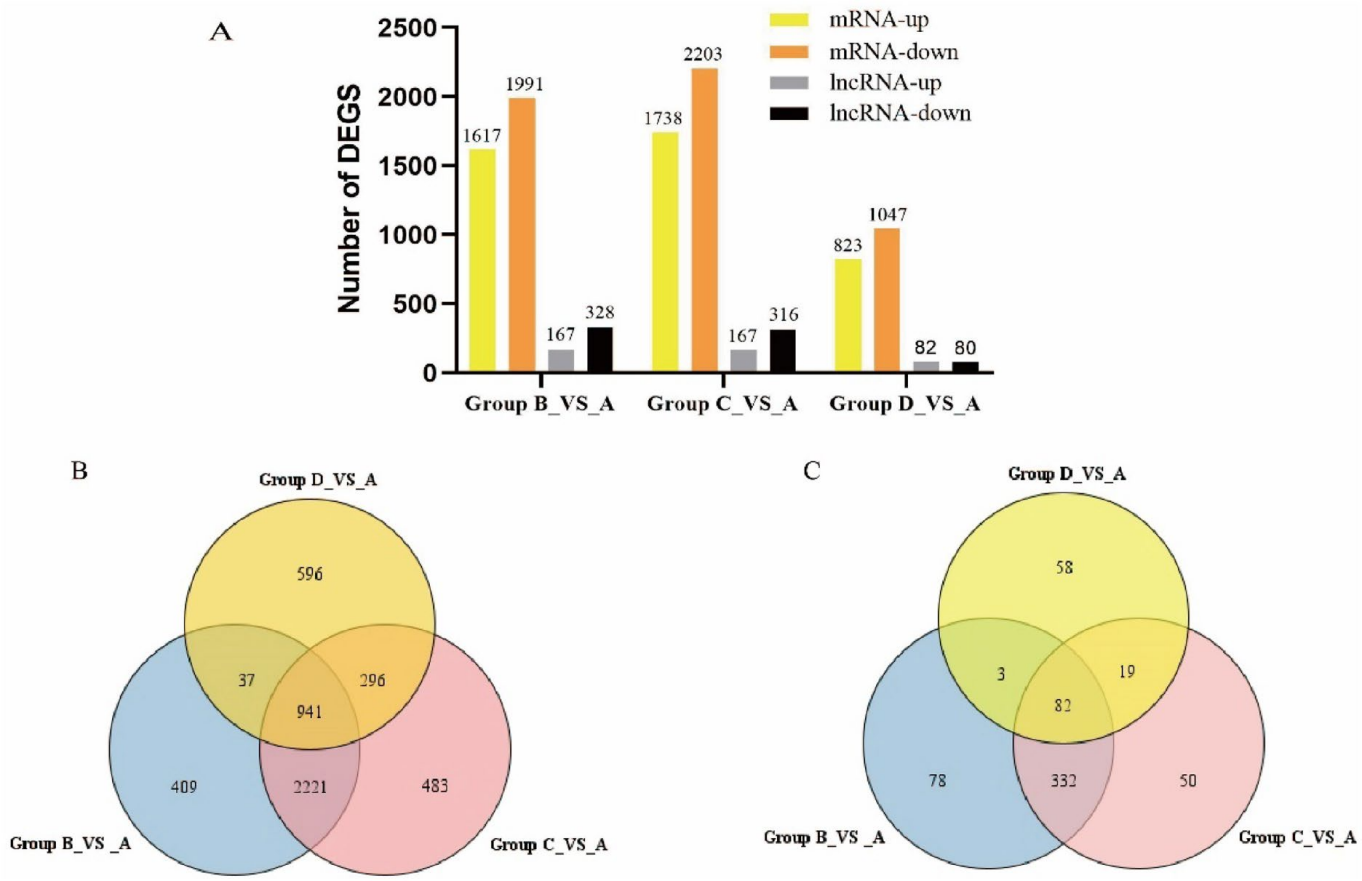
Number of differentially expressed lncRNAs and mRNAs and Venn diagram showing the number of overlapping DE-lncRNAs and DE-mRNAs in three groups. (**A**) DE-lncRNAs and DE-mRNAs (**B**) Venn diagram of mRNAs. (**C**) Venn diagram of lncRNAs.

We performed DE analyses between the storage groups and the fresh groups using FPKM. Based on the criteria of |log2FoldChange|≥ 1 and FDR < 0.05, there were 495, 483, 162 DE-lncRNAs and 3680, 3941, 1870 DE-mRNAs in group B, group C, and group D compared to group A, respectively. Both group B and group C showed more numbers of DEGs than group D. Moreover, except for lncRNA in group D, downregulated genes of mRNA and lncRNA in group A and group B were higher than upregulated genes (Fig. 2A).

Thereafter, we performed Venn analyses for the DE lncRNAs and DE mRNAs of three groups. We found 82 common lncRNAs and 941 common mRNAs differentially expressed throughout nearly the entire postharvest storage (Fig. 2B,C). Of 82 DE common lncRNAs, thirty-three were upregulated and forty-seven were downregulated. The other two lncRNAs (XLOC_003962, XLOC_008798) were downregulated in group B and group C but were upregulated in group D. Of 941 DE common mRNAs, three hundred and seventy-six were upregulated, and five hundred and fifty were downregulated. Among the remaining 15 DE common mRNAs, 14 were downregulated in group B and group C, and upregulated in group D, while 1 was upregulated in group B and group C, and downregulated in group D.

### Differential expression of lncRNAs target gene predicition

The cis- and trans-targets of the DE-lncRNAs in group B, C and D were predicted, respectively. Next, we focused on co-identified DE-lncRNAs in all three groups and forecasted the cis- and trans-targets of these DE-lncRNAs. We identified 205 lncRNA-mRNA pairs, which lncRNAs acted in cis with mRNA. Among the 205 lncRNA-mRNA pairs, there were 93 positive correlated pairs, 102 negative correlated pairs, and 10 overlapping pairs. For the trans-targets of co-identified DE-lncRNAs, 12,257 lncRNA-mRNA pairs were identified.

### Gene ontology (GO) analysis of DE-lncRNAs target gene

**Figure 3:**
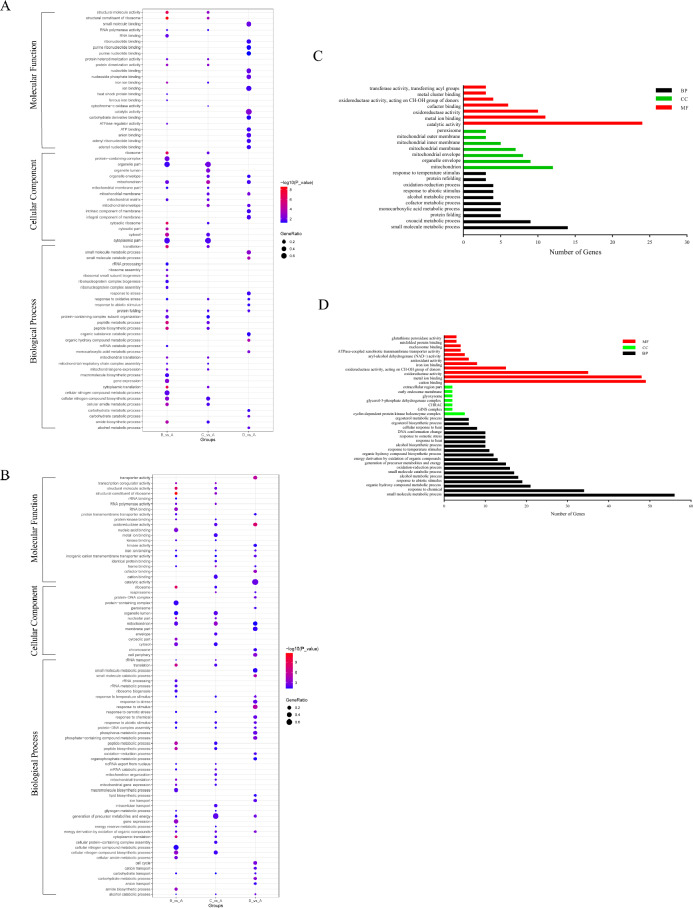
GO analysis of differentially expressed target mRNAs of DE-lncRNAs. (**A**) GO analysis of the DE-target mRNAs regulated in cis by DE-lncRNAs (**B**) GO analysis of the DE-target mRNAs regulated in trans by DE-lncRNAs (**C**) GO analysis of the DE-target mRNAs regulated in cis by co-identified DE-lncRNAs (**D**) GO analysis of the DE-target mRNAs regulated in trans by co-identified DE-lncRNAs.

GO analysis of the DE-target mRNAs regulated in cis by DE-lncRNAs enriched 165, 198, 235 significantly terms (*P* < 0.05) in group B, C, and D, respectively. 217, 225 and 393 significantly GO terms (*P* < 0.05) in group B, C, and D were enriched across the DE-target mRNAs regulated in trans by DE-lncRNAs. These GO terms were classified into three different groups as biological process, molecule function and cellular component. These GO terms with a high generatio and a p value of less than 0.05 in three groups were shown (Fig. 3A,B). As showed in the Fig. 3A,B, we found that the main biological processes in group B and C included peptide metabolic and biosynthetic process, and translation. While in group D, the main biological processes were small molecule metabolic process, carbohydrate metabolic process and alcohol metabolic process. Response to abiotic stimulus was enriched throughout the entire postharvest storage.

Go analysis of the DE-target mRNAs regulated in cis by co-identified DE-lncRNAs in union three groups enriched 155 significantly terms (*P* < 0.05). These terms included 70 biological processes, 56 molecular functions, and 29 cellular components and primarily involved in catalytic activity, small molecule metabolic process and response to stimulus. We identified 265 GO terms significantly enriched across the DE-target mRNAs regulated in trans by co-identified DE-lncRNAs in union three groups. These terms included 179 biological processes, 60 molecular functions, and 26 cellular components. Among 265 GO terms, the pathways including alcohol metabolic process, oxidoreductase activity, response to abiotic stimulus, and ergosterol metabolic process may play a crucial role in postharvest senescence of *S. latifolia* (Fig. 3C,D).

### KEGG pathway analysis of DE-lncRNAs target genes

**Figure 4:**
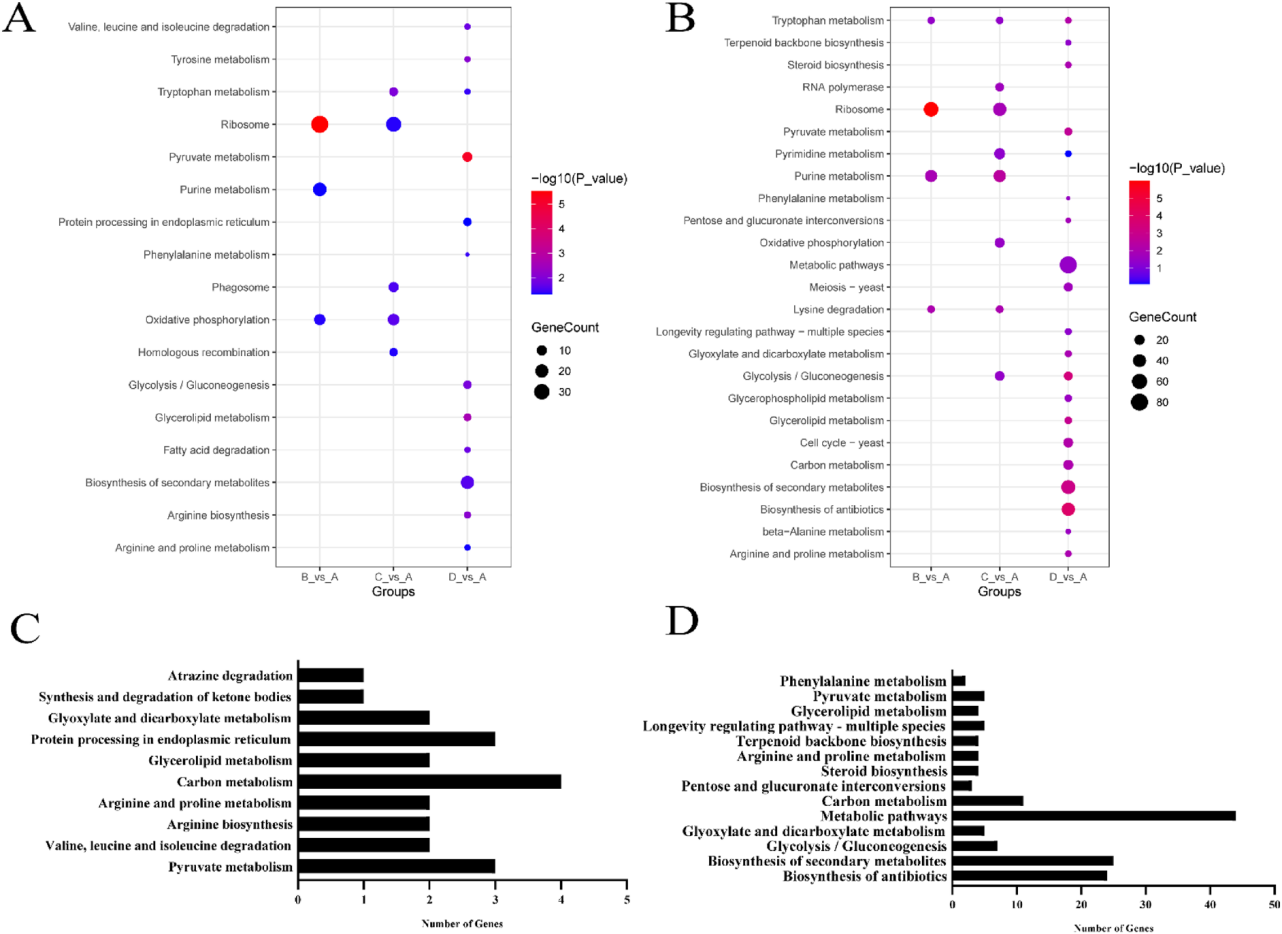
KEGG analysis of differentially expressed target mRNAs of DE-lncRNAs. (https://www.genome.jp/kegg/pathway.html) (**A**) KEGG analysis of the DE-target mRNAs regulated in cis by DE-lncRNAs (**B**) KEGG analysis of the DE-target mRNAs regulated in trans by DE-lncRNAs (**C**) KEGG analysis of the DE-target mRNAs regulated in cis by co-identified DE-lncRNAs (**D**) KEGG analysis of the DE-target mRNAs regulated in trans by co-identified DE-lncRNAs.

We performed KEGG enrichment analysis of DE-lncRNAs target genes for three groups (B, C, D) (Fig. 4A,B). The DE-target mRNAs of DE-lncRNAs regulated in cis were enriched in 95, 100, and 67 KEGG pathways in group B, C and D, respectively. The results showed that three pathways including ribosome, oxidative phosphorylation, and purine metabolism were significantly enriched in group B; five pathways including tryptophan metabolism, oxidative phosphorylation, phagosome, ribosome, and homologous recombination were significantly enriched in group C; twelve pathways were significantly enriched in group D including pyruvate metabolism, glycerolipid metabolism, arginine biosynthesis, etc. (*P* < 0.05). We also performed KEGG analysis of the DE-target mRNAs regulated in trans by DE-lncRNAs. The results showed 107 pathways were enriched in group B, four of which including ribosome, lysine degradation, purine metabolism, tryptophan metabolism was significantly enriched. 100 pathways were enriched in group C, eight of which including ribosome, lysine degradation, purine metabolism etc. were significantly enriched. 93 pathways were enriched in group D, 19 of which including biosynthesis of antibiotics, glycolysis gluconeogenesis, biosynthesis of secondary metabolites, pyruvate metabolism etc. were significantly enriched.

Meanwhile, we also performed KEGG enrichment analysis of co-identified DE-lncRNAs target genes. The DE-target mRNAs regulated in cis by co-identified DE-lncRNAs in union three groups enriched 11 significantly terms (*P* < 0.05). The DE-target mRNAs regulated in trans by co-identified DE-lncRNAs in union three groups enriched 14 significantly terms. These terms mainly included carbon metabolism, amino acid metabolism and pyruvate metabolism (Fig. 4C,D).

### LncRNA-mRNA interaction network

**Figure 5:**
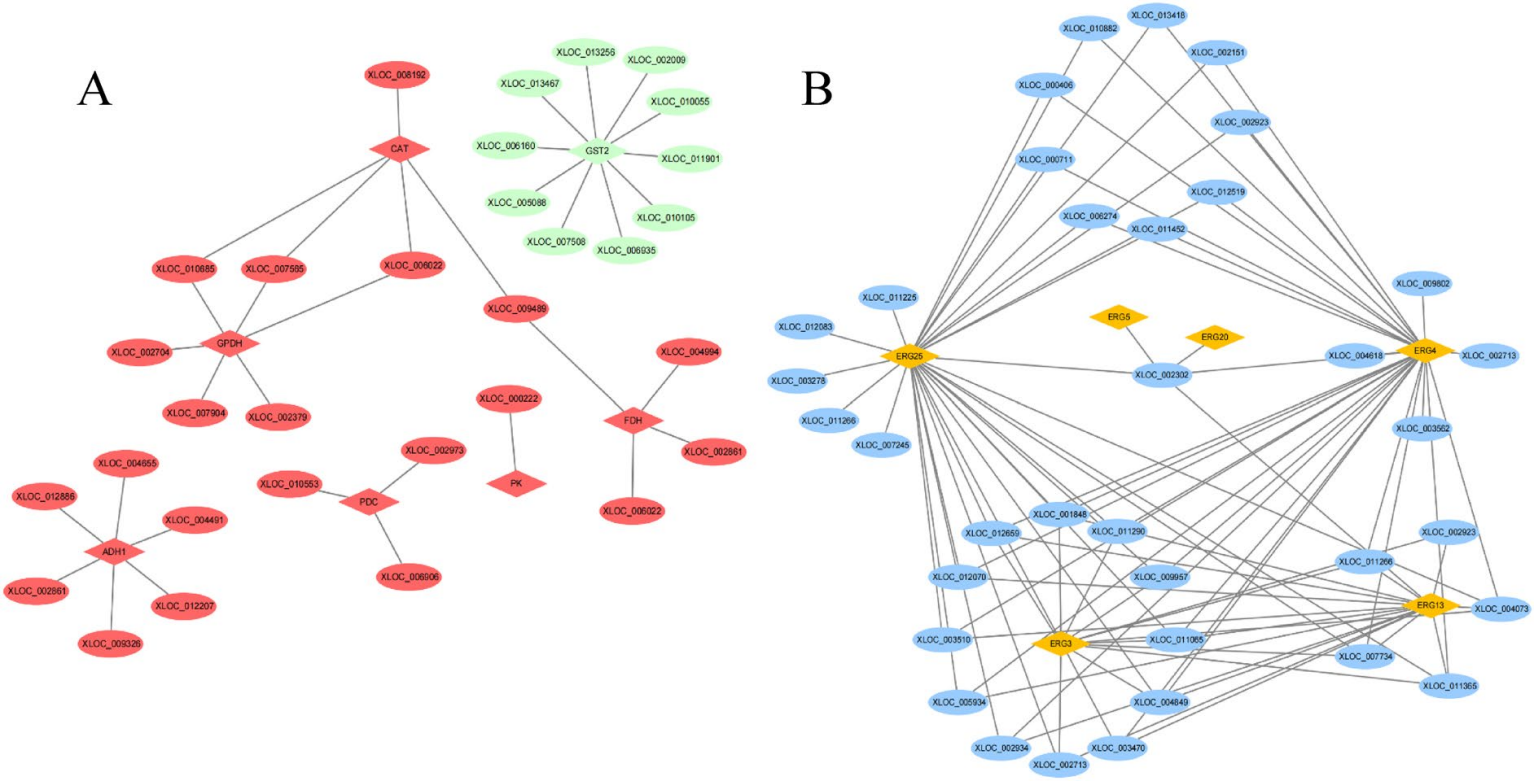
Interaction network between DE-lncRNAs and DE-mRNAs.

The possible regulatory network interactions were forecasted in this study. We analyzed the DE-lncRNAs and DE-mRNAs from union three groups involved in ergosterol biosynthetic and metabolic process, oxidoreductase activity, alcohol metabolic process etc. pathways and constructed their networks (Fig. 5). We found that 11 lncRNAs interacted with 3 mRNAs in the oxidoreductase activity pathway (Fig. 5A), while 36 lncRNAs interacted with 6 mRNAs in the ergosterol metabolic process pathway (Fig. 5B).

### Validation of DE-lncRNAs and mRNAs by RT-qPCR

**Figure 6:**
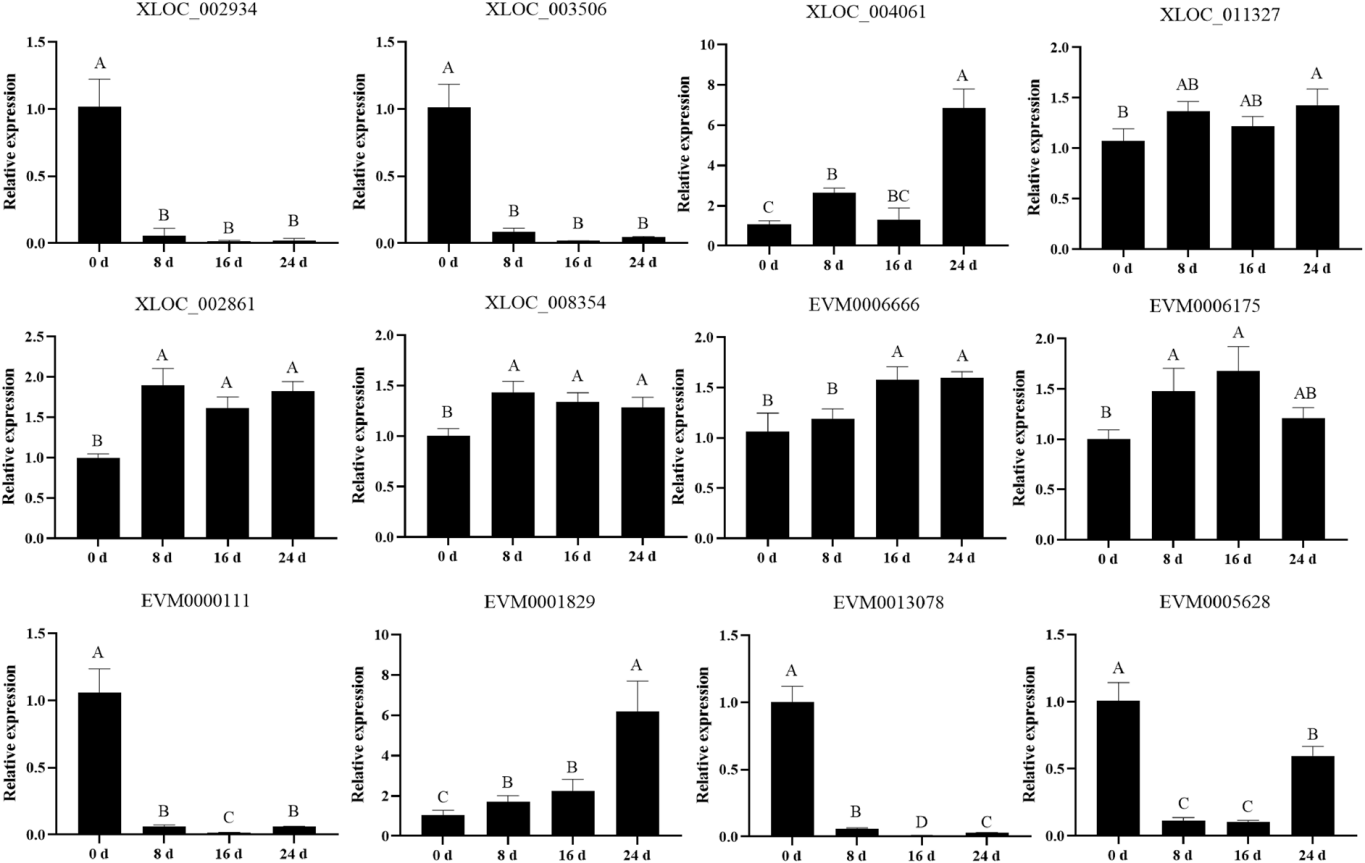
RT-qPCR validation of differentially lncRNAs and mRNAs.

To validate the high-throughput sequencing results, six lncRNAs (XLOC_002934, XLOC_003506, XLOC_004061, XLOC_011327, XLOC_002861, XLOC_008354) and six mRNAs (EVM0000111, EVM000013078, EVM0001829, EVM0006666, EVM0006175, EVM0005628) were selected to analyze the expression levels using RT-qPCR (Fig. 6). The results showed that the expression patterns of chosen DE-lncRNAs and DE-mRNAs were consistent with RNA-seq. XLOC_002934, XLOC_003506, EVM0000111, EVM000013078 and EVM0005628 were all down-regulated at 8, 16, and 24 days. XLOC_004061, XLOC_011327, XLOC_002861, XLOC_008354, EVM0006175, EVM0006666 and EVM0001829 were all up-regulated at 8, 16, and 24 days. These results confirmed the reliability of the RNA-seq sequencing data.

## Discussion

No matter plants, crops or mushrooms, they would suffer from oxidative stress^24^, deficient energy^25^, and microbial attack^26^ after harvest. It's reported that when plants progress into the stage of senescence, they usually initiate stress-resistant mechanism^27^. Increasing evidence suggests that lncRNAs are contribute to both biotic and abiotic stresses resistance^16,28,29^. So, many lncRNAs have been identified in human, animal, plants, and crops, and the regulatory mechanisms have been explored^20,30^. For example, DE-lncRNAs were identified as responses to thermal and hypoxic stresses in sea cucumber^31^. A few DE-lncRNAs were also identified in tomato roots which respond positively to salt stress ^32^. However, what is not yet clear in the importance of lncRNAs in postharvest senescence of *S. latifolia*. As far as we know, information concerning the lncRNAs expression data and function in *S. latifolia* remains lacking. This is the first systematic identification of lncRNAs in *S. latifolia*. *S. latifolia* is a good model fungal species because of its special growth characteristics and economic value^33^. LncRNAs of *S. latifolia* have fewer exon numbers and shorter lengths than mRNAs which were in agreement with previous studies on tomato^32^, peanut^34^ and arabidopsis^15^. Meanwhile, these lncRNAs have a remarkable characteristic in this study that the highly expressed lncRNAs were more than mRNAs. This characteristic may suggest lncRNAs have crucial roles in stresses resistance in *S. latifolia* and need to be investigated.

We identified DE-lncRNAs and DE-mRNAs between the fresh and storage groups, and there were 82 common DE-lncRNAs and 941 common DE-mRNAs throughout nearly the entire postharvest storage. In this study, some of these DE-lncRNAs and their target genes have been confirmed to play essential roles in aging process. LncRNAs regulated their target genes expression through a cis- or trans-mediated process. We performed functional analyses of these target genes (Figs. 3 and 4). The results of GO and KEGG pathway analyses could help us to understand the mechanisms of postharvest senescence of *S. latifolia.* Numerous age-related genes and pathways of *Volvariella volvacea* were screened using GO and KEGG analyses in different storage period^11^. In this study, some of the same aged-related pathways were found. Amino acid biosynthesis and metabolism, such as “valine, leucine and isoleucine degradation”, “arginine and proline metabolism”, “phenylalanine metabolism”, and “arginine biosynthesis” were significantly enriched. Protein synthesis and transcription, such as “protein processing in endoplasmic reticulum”, “ribosome” and “protein folding” were significantly enriched. Energy metabolism, such as “carbon metabolism”, “NADH oxidation”, “mitochondrion” and “ATPase-coupled xenobiotic transmembrane transporter activity” were significantly enriched. Other age-related pathways, such as “alcohol metabolic process”, “oxidation–reduction process”, “cellular response to stress”, and “ergosterol metabolic process” were also significantly enriched. We sorted out some key genes and corresponding lncRNAs (Table 2) in the pathway to discuss below.

**Table 2:** Long non-coding RNAs (lncRNAs) and lncRNA target genes that are associated with postharvest senescence.

Gene_id	Gene_indentification	Cis-lncRNAs	Trans-lncRNAs
EVM0012176	Formate dehydrogenase (FDH)	XLOC_002861	XLOC_006022, XLOC_009489, XLOC_004994
EVM0007108	Alcohol dehydrogenase 1 (ADH1)		XLOC_004491, XLOC_004655, XLOC_002861, XLOC_012207, XLOC_012886, XLOC_009326
EVM0009327	Pyruvate decarboxylase (PDC)		XLOC_010553, XLOC_006906, XLOC_002973
EVM0011998	catalase (CAT)		XLOC_008192, XLOC_007565, XLOC_010685, XLOC_009489, XLOC_006022
EVM0000573	Glutathione S-transferase 2 (GST2)		XLOC_005088, XLOC_007508, XLOC_013467, XLOC_006935, XLOC_010055, XLOC_010105, XLOC_006160, XLOC_002009, XLOC_011901, XLOC_013256
EVM0008918	Glycerol-3-phosphate dehydrogenase (GPDH)		XLOC_002379, XLOC_006022, XLOC_007904, XLOC_002704, XLOC_010685, XLOC_007565
EVM0009831	Pyruvate kinase (PK)		XLOC_000222
EVM0000111	Methylsterol monooxygenase (ERG25)		XLOC_011266, XLOC_004073, XLOC_011365, XLOC_007734, XLOC_011065, XLOC_004849, XLOC_003470, XLOC_002713, XLOC_002934, XLOC_002923, XLOC_005934, XLOC_003510, XLOC_012519, XLOC_012070, XLOC_011452, XLOC_012659, XLOC_006274, XLOC_001848, XLOC_011290, XLOC_000711, XLOC_009957, XLOC_000406, XLOC_007245, XLOC_010882, XLOC_003278, XLOC_012083, XLOC_013418, XLOC_011225, XLOC_002151
EVM0003490	Delta(24(24(1)))-sterol reductase (ERG4)		XLOC_002713, XLOC_004073, XLOC_003470, XLOC_012659, XLOC_011065, XLOC_005934, XLOC_002934, XLOC_007734, XLOC_011266, XLOC_012070, XLOC_004849, XLOC_003510, XLOC_000711, XLOC_006274, XLOC_011365, XLOC_002923, XLOC_012519, XLOC_011452, XLOC_010882, XLOC_000406, XLOC_013418, XLOC_001848, XLOC_003562, XLOC_002151, XLOC_011290, XLOC_004618, XLOC_009957, XLOC_009802
EVM0005394	Hydroxymethylglutaryl-CoA synthase (ERG13)	XLOC_002302	XLOC_002923, XLOC_011266, XLOC_002713, XLOC_012659, XLOC_004073, XLOC_004849, XLOC_003470, XLOC_012070, XLOC_007734, XLOC_011065, XLOC_011365, XLOC_005934, XLOC_002934, XLOC_001848, XLOC_003510
EVM0012147	Delta(7)-sterol 5(6)-desaturase (ERG3)		XLOC_002923, XLOC_011266, XLOC_011290, XLOC_001848, XLOC_004849, XLOC_012659, XLOC_004073, XLOC_011065, XLOC_007734, XLOC_002713, XLOC_009957, XLOC_011365

Pyruvate kinase (PK) is one of the rate-limiting enzymes of glycolysis that catalyzes the final reaction of phosphoenolpyruvate (PEP) and ADP to pyruvate and ATP^35^. High expression of PK would contribute to pyruvate accumulation which could protect fungal cells from ROS damage under heat stress^36^. The product pyruvate feeds into a series of metabolism pathways like ethanol fermentation under hypoxic stress. In postharvest storage of *S. latifolia*, PK was significantly induced, 1 lncRNAs (XLOC_000222) might mediate carbohydrate metabolic process by trans-regulating PK expression. Alcohol dehydrogenase (ADH) and pyruvate decarboxylase (PDC) are key enzymes in ethanol fermentation. PDC decomposed pyruvate to acetaldehyde. ADH reduced acetaldehyde to ethylalcohol. It’s reported that ADH and PDC play important roles in responding to senescence stress^37^. When the oxygen was consumed to a relatively low level in the OPP film, *S. latifolia* would maintain its energy demand by alcohol fermentation. ADH1 (EVM0007108) and PDC (EVM0009327) were both up-regulated throughout the entire postharvest storage and could be regulated by 6 (XLOC_004491, XLOC_004655, XLOC_002861, XLOC_012207, XLOC_012886 and XLOC_009326) and 3 lncRNAs (XLOC_010553, XLOC_006906, XLOC_002973) in trans, respectively. This suggested that these 9 lncRNAs might play a role in alcohol metabolic process pathway by targeting ADH1 and PDC under senescence stress. Formate dehydrogenases (FDH) are a group of enzymes that catalyze the oxidation of formate to carbon dioxide, which are also involved in energetic metabolism and pathways related to stress response^38^. The FDH activity increased significantly with increasing copper concentrations to help cotton scope with copper toxicity^39^. Under hypoxic stress, FDH activity is significantly increased in the potato tubers^40^. FDH (EVM0012176), which was cis-regulated by 1 lncRNAs (XLOC_002861) and trans-regulated by 3 lncRNAs (XLOC_006022, XLOC_009489, XLOC_004994), was significantly up-regulated during the entire postharvest storage of *S. latifolia.* This indicated that FDH could be induced under low oxygen condition to supply *S. latifolia* with additional energy.

Reactive oxygen species (ROS) are produced during postharvest storage, which can damage proteins, DNA, and lipids, resulting in senescence^41^. Scavenging ROS is important for organism and there are antioxidant mechanisms in cell including enzymatic and non-enzymatic reactions^42^. Superoxide dismutase (SOD), catalase (CAT), and glutathione s-transferase (GST) are main enzymes in enzymatic reaction, and biomarkers of oxidative stress^43^. In this study, higher level of GST 2 (EVM0000573) and CAT (EVM0011998), which were trans-regulated by 10 lncRNAs (XLOC_005088, XLOC_007508, XLOC_013467, XLOC_006935, XLOC_010055, XLOC_010105, XLOC_006160, XLOC_002009, XLOC_011901, XLOC_013256) and 5 lncRNAs (XLOC_008192, XLOC_007565, XLOC_010685, XLOC_009489, XLOC_006022), respectively, were accumulated during the entire postharvest storage. This means that these 15 lncRNAs might be involved in antioxidant activity pathway through transaction on GST 2 and CAT.

Glycerol-3-phosphate dehydrogenase (GPDH) gene family are widely known to be involved in response to various stresses in different species^44–47^. The expression of GPDH (EVM0008918) was significantly induced and predicted to be trans-targeted by 6 lncRNAs (XLOC_002379, XLOC_006022, XLOC_007904, XLOC_002704, XLOC_010685, XLOC_007565) during the entire postharvest storage of *S. latifolia.* GPDH were involved in multiple metabolic pathways such as alcohol metabolic process, NADH metabolic process, energy derivation by oxidation of organic, oxidation–reduction process, cellular carbohydrate metabolic process and so on. This also indicated that GPDH could help to supply energy and maintain redox balance.

In addition to these, ergosterol biosynthesis and metabolism are very important in adaptation to stress^48^. For example, the decrease in ergosterol abundance is an adaptive response to hyperosmotic and oxidative stress^49^. Usually, ergosterol in fungi is synthesized through many highly conserved steps that many biosynthetic enzymes are involved^50,51^. Thus, one of the ways to regulate of ergosterol biosynthetic (ERG) is feedback inhibition of enzymes^48^. In this study, 6 genes (EVM0000111, EVM0000659, EVM0003490, EVM0004880, EVM0005394, EVM0012147) encoding the ergosterol specific enzymes of the ERG pathway were all significantly down-regulated, 4 (EVM0000111, EVM0003490, EVM0005394, EVM0012147) of which could be regulated by lncRNAs (Table 2). This suggested that these lncRNAs might play a role in the ERG pathway by targeting ERG25, ERG4, ERG13 and ERG3 during postharvest fruiting body of *S. latifolia* senescence.

In summary, we reported the first lncRNAs expression profiles of *S. latifolia* and found that lncRNAs might influence the aging process by affecting target genes. Through analyzing the function of lncRNA target genes, some target genes corresponding to pathways played important roles in *S. latifolia* response to aging stress. The pathway categories mainly included carbon and energy metabolism, response to abiotic stimulus (temperature, oxidative stress and osmotic stress), amino acid biosynthesis and metabolism, and protein synthesis and transcription. We also comprehensively analyzed the DE-lncRNA-mRNA co-expression networks in response to aging stress. All these findings provide novel insights into the better understanding of lncRNAs regulatory role in the postharvest senescence of *S. latifolia* packed with OPP film during 4 ℃ storage, and could also provide useful information for improving preservation method of *S. latifolia.*

## Materials and methods

### Experimental sample

The fresh fruiting bodies of *S. latifolia* (strain, Minxiu No.1) were harvested from Fujian TIANYI Mushrooms Co., Ltd., China, and transported to the laboratory within 2 h under refrigerated conditions after picking. Then, they (50 ± 5 g) were screened for uniform color and absence of mechanical damage, packaged in oriented polypropylene (OPP) film (25 μm thickness, size 20 cm × 18 cm). After that, they were randomly divided into four groups and stored at 4 ℃ for 24 d (Group A: 0 day; Group B: 8 days; Group C: 16 days; Group D: 24 days). Subsequently every 8 days, three replicates were frozen with liquid nitrogen and stored at − 80 ℃ for RNA analysis.

### Library preparation and RNA-sequence

Total RNA was extracted from *S. latifolia* samples using the TRIzol reagent. Then, the quantity and quality of total RNA were examined. Ribosomal RNA was removed using Epicentre Ribo-Zero™ Gold Kits (Human/Mouse/Rat/other) (Epicentre, USA). Subsequently, the sequencing libraries were generated following manufacture commendations with varied index label by NEBNext^®^ Ultra™ Directional RNA Library Prep Kit for Illumina (NEB, Ispawich, USA). The libraries were sequenced on an Illumina HiseqX platform.

### Quality control, alignment

The original sequencing data contains adapter sequences and low-quality sequences. In order to ensure the quality of data, Cutadapt (V 2.7) was used to filter the original sequences and trim the reads with low sequencing quality. Finally, high-quality clean data were gotten. Fastqc (v 0.11.8) was used for data statistics.

HISAT2 (v 2.1.0) was used to align clean reads to the reference sequence (https://www.ncbi.nlm.nih.gov/genome/62827?genome_assembly_id=1624781). RSeQC (V 3.0.1) was used to evaluate the quality of the comparison results of sequencing, which mainly included sequencing saturation, gene coverage and the distribution of reads in different regions of the reference genome.

### Transcriptome assembly and lncRNAs identification

To find previous unannotated transcription information and discover new transcripts and new genes, we used StringTie to assemble the mapped reads and compared them with previous genome annotation (provided by Yang Chi)^4^. Then we adapted three steps of screen and filtration to identify lncRNAs from new transcripts: (1) transcripts those with lengths were > 200 nt, exons > 2 and ORF length < 300 nt were retained; (2) compared with *S. latifolia* annotation file and retained transcripts whose class_code was i, j, u, o, x. (3) three software programs, CPAT, CNCI, and PLEK were used to assess the protein-coding potential and removed all transcripts with known protein-coding capability. After the above screening, the last retaining transcripts were considered as lncRNAs^52^.

### Quantitation of gene expression levels and differential gene expression analysis

StringTie (v 1.3.3b) was used to evaluate the expression levels of mRNA and lncRNA, respectively. StringTie calculated the FPKM value of each gene/transcript in the sample according to the comparison results of Hisat2 software. Gene differential expression analysis were assayed by DESeq2 (R package V 1.24.0). Differential gene screening mainly refers to the factor of difference (Fold change value) and FDR value (padj value, Pvalue value after correction) as related indicators. The differential gene with |log2 Fold change|≥ 1 and FDR < 0.05 was selected as the significant difference gene^31^.

### Target gene prediction

LncRNAs do not encode protein and are mainly achieved by acting on protein-coding genes by cis or trans. Therefore, cis and trans target analysis were performed, respectively. Functions of the differentially expressed LncRNAs were indirectly predicted through the target genes. Those mRNAs within the range of 10 kb upstream and downstream of each lncRNAs were identified as cis-target genes. The cor function in R was used to analyze the expression relationship between LncRNAs and mRNAs pairs. When the value of cor ≥ 0.85, those mRNAs were identified as trans-target genes^14^. The lncRNA-mRNA interaction networks were constructed using Cytoscape (Table S1).

### GO and KEGG enrichment analyses of DE-lncRNAs target genes

For GO analyses, protein sequences of Swissport were downloaded from Uniport database (https://www.uniprot.org/); picked out the protein sequence of model species *Saccharomyces cerevisiae* for alignment and then corresponded to the GO functional annotation results in Swissport (http://www.expasy.ch/sprot/).

For KEGG analyses, compared with the protein sequences of *Saccharomyces cerevisiae* in KEGG database (http://www.genome.jp/kegg/) and mapped to the pathway results of KEGG of *Saccharomyces cerevisiae*^53,54^.

### Real-time quantitative PCR (RT-qPCR) validation

To verify gene expression, several genes were chosen for RT-qPCR. Total RNA was extracted and then the quality and concentration of RNA was measured. The first-strand cDNA was synthesized using Hifair^®^ III 1st Strand cDNA Synthesis SuperMix for qPCR (YEASEN). Gene-specific primers were designed using Primer 3 (Table S1). A SYBR Green^®^ real-time PCR assay (YEASEN) was used for examining the gene expression levels and GAPDH was used as the internal control of *S. latifolia*. Relative gene expression levels were analyzed using the 2^−△△CT^ method^33^.

### Supplementary Information

Supplementary Table S1.

## Data Availability

The data of RNA-seq in this study has been deposited in NCBI’s Gene Expression Omnibus (GEO) under accession number GSE226806.
