# Supplementary material for: Long non-coding RNAs and their potential function in response to postharvest senescence of Sparassis latifolia during cold storage
Source: Sci Rep. 2024 Jan 7;14:747. doi: 10.1038/s41598-023-46744-2 (PMC10772075; doi:10.1038/s41598-023-46744-2)
Supplement: Supplementary file 1 — Supplementary Table S1. [file 41598_2023_46744_MOESM1_ESM.pdf]

Long non-coding RNAs and their potential function in response to postharvest senescence of *Sparassis latifolia* during cold storage

Mengting Weng[1,2], Di Zhang[1,2], Hongyu Wang[1,2], Chi Yang[1,2], Hongyi Lin[1,2], Yanfang Pan[3,*], Yanquan Lin[1,2,*]

[1]Institute of Edible Mushroom, Fujian Academy of Agricultural Sciences, Fuzhou 350014, China

[2]National and Local Joint Engineering Research Center for Breeding & Cultivation of Featured Edible Mushroom, Fujian Academy of Agricultural Sciences, Fuzhou 350014, China

[3]Institute of Food Science and Technology, Chinese Academy of Agricultural Sciences, Beijing 100193, China

Corresponding author:

panyanfang13@126.com

lyq-406@163.com (YQ.L.)

Supplementary Table 1

13 Genes in *Sparassis latifolia* Used for qRT-PCR Analysis

Gene	Primer sequence (5'-3')
GAPDH	F: TCATTACCGCACCCTCTTCC
	R: CCACCACGCCAGTCTTTATG
XLOC_002934	F:CTGGTCGGAAACCAAGGTGA
	R:AAAAGAATCTCGGCCGTCGT
XLOC_003506	F:GCGATCACGAAACTCGAAGC
	R:TTCCGGCGAATGTATCGGAG
XLOC_004061	F:CGTACCACCCAGCTTGACTT
	R:GGGGTATGGCAGAGCAACTT
XLOC_011327	F:GGGTCGTCATCCTCGTTAGC
	R:TGGGCTGCGGGATATTGTTT
XLOC_008354	F:ACCACCACATTCACATCGCT
	R:GTGCTCTGAGGCACTCCTAC
XLOC_002861	F:CGCCCTCCAGTGTATTGGTT
	R:AGGGCCTTAGATGCTCCTGA
EVM0000111	F:TCCTCGGACCATTGGGGTAT
	R:TGCATGAAATGGCTCCCACT
EVM0006666	F:CGTTATCCTCACGCGATCCA
	R:TGGACCTGAAGAATCCGTGC
EVM0006175	F:TTCGTGCTGCATTCCGATCT
	R:TCCCACTCCTGAGTTCGCTA
EVM0001829	F:TACCAGCAACCCGTCAACTC
	R:CGTGTTTGTGTCGGTGGTTC
EVM0005628	F:GGATCCGCAGGTATGGAAGG
	R:CCTTGCTCACAAAGCCAACC
EVM0013078	F:GGCTTCTACCGGATCTGAGC
	R:AAGCCAGTTGTCGTGTCGAT
